# Radiation exposure and screening yield by digital breast tomosynthesis compared to mammography: results of the TOSYMA Trial breast density related 

**DOI:** 10.1007/s00330-024-10847-9

**Published:** 2024-07-16

**Authors:** Alexander Sommer, Stefanie Weigel, Hans-Werner Hense, Joachim Gerß, Veronika Weyer-Elberich, Laura Kerschke, Elke Nekolla, Horst Lenzen, Walter Heindel, for the TOSYMA Screening Trial Study Group, Walter Heindel, Stefanie Weigel, Joachim Gerß, Hans-Werner Hense, Gerold Hecht, Alexander Sommer, Horst Lenzen, Jörg Czwoydzinski

**Affiliations:** 1 Clinic for Radiology and Reference Center for Mammography Münster, University of Münster and University Hospital Münster, Münster, Germany; 2 Institute of Epidemiology and Social Medicine, University of Münster, Münster, Germany; 3 Institute of Biostatistics and Clinical Research, University of Münster, Münster, Germany; 4 Federal Office for Radiation Protection, Department of Medical Radiation Protection, Neuherberg, Germany; 5 Reference Center for Mammography North, Oldenburg, Germany

**Keywords:** Mammography screening, Radiation exposure, Breast density, Digital breast tomosynthesis, Breast cancer detection

## Abstract

**Objectives:**

The randomized TOmosynthesis plus SYnthesized MAmmography (TOSYMA) screening trial has shown that digital breast tomosynthesis plus synthesized mammography (DBT + SM) is superior to digital mammography (DM) in invasive breast cancer detection varying with breast density. On the other hand, the overall average glandular dose (AGD) of DBT is higher than that of DM. Comparing the DBT + SM and DM trial arm, we analyzed here the mean AGD and their determinants per breast density category and related them to the respective invasive cancer detection rates (iCDR).

**Methods:**

TOSYMA screened 99,689 women aged 50 to 69 years. Compression force, resulting breast thickness, the calculated AGD obtained from each mammography device, and previously published iCDR were used for comparisons across breast density categories in the two trial arms.

**Results:**

There were 196,622 exposures of 49,227 women (DBT + SM) and 197,037 exposures of 49,132 women (DM) available for analyses. Mean breast thicknesses declined from breast density category A (fatty) to D (extremely dense) in both trial arms. However, while the mean AGD in the DBT + SM arm declined concomitantly from category A (2.41 mGy) to D (1.89 mGy), it remained almost unchanged in the DM arm (1.46 and 1.51 mGy, respectively). In relative terms, the AGD elevation in the DBT + SM arm (64.4% (A), by 44.5% (B), 27.8% (C), and 26.0% (D)) was lowest in dense breasts where, however, the highest iCDR were observed.

**Conclusion:**

Women with dense breasts may specifically benefit from DBT + SM screening as high cancer detection is achieved with only moderate AGD elevations.

**Clinical relevance statement:**

TOSYMA suggests a favorable constellation for screening with digital breast tomosynthesis plus synthesized mammography (DBT + SM) in dense breasts when weighing average glandular dose elevation against raised invasive breast cancer detection rates. There is potential for density-, i.e., risk-adapted population-wide breast cancer screening with DBT + SM.

**Key Points:**

- *Breast thickness declines with visually increasing density in digital mammography (DM) and digital breast tomosynthesis (DBT).*
- *Average glandular doses of DBT decrease with increasing density; digital mammography shows lower and more constant values.*
- *With the smallest average glandular dose difference in dense breasts, DBT plus SM had the highest difference in invasive breast cancer detection rates.*

## Introduction

Breast cancer is the leading cause of cancer morbidity and mortality in women worldwide [1]. Mammography screening is an effective tool for early breast cancer detection and has been proven to reduce the number of deaths by breast cancer [2, 3]. The current standard in population-based mammography screening programs (MSPs) is full-field digital mammography (DM). Breast cancer detection in DM-based screening programs, however, might be limited in women with extremely dense breast parenchyma: while these women are known to have an increased risk of breast cancer [4], radiological summation and superposition effects in dense breasts can significantly compromise cancer detection in DM examinations by masking radiological cancer signs [5–7]. A technical mammography advancement, digital breast tomosynthesis (DBT), reduces lesion masking from adjacent breast tissue by acquiring multiple low-dose images with varying angles by an arch-like movement of the tube and reconstruction of thin DBT slices. In addition, a two-dimensional mammographic image, labeled a synthesized mammogram (SM), can be reconstructed from the pseudo-3-dimensional DBT data set, e.g., for comparison with previous DM images and assessment of breast density [8, 9].

The large randomized controlled trial TOSYMA (TOmosynthesis plus SYnthesized MAmmography) showed that, in a screening of women aged 50 to 69 years, DBT + SM leads to a significant increase by 48% of the invasive breast cancer detection rates (iCDR) compared to standard DM screening [10]. Moreover, a subsequent subanalysis of the TOSYMA data revealed higher iCDR with DBT + SM than DM, especially in women with extremely dense breast tissue [11, 12].

Of note, however, the overall average glandular dose (AGD) was higher in the DBT + SM arm than in the DM arm [10]. Therefore, the anticipated benefits of DBT + SM screening have to be weighed against the risks from increased radiation exposure required with different breast densities. The large TOSYMA screening trial, embedded in a population-wide screening program, produced ample individual radiation exposure data of women screened with DBT + SM in comparison to DM. This trial appeared particularly suitable to generate a more detailed understanding of the radiation doses applied with the two screening techniques. Therefore, the aim of this TOSYMA subanalysis was to compare the AGD applied in women from the two trial arms, stratified by mammographic breast density categories, and to relate this to the detection rates of invasive breast cancers.

## Materials and methods

### Trial design and recruitment

TOSYMA is a prospective, randomized, controlled multicentre trial, which compared women subjected to a DBT + SM or a DM screening. It was embedded within the population-wide German MSP that biennially invites women, aged 50 to 69 years, to a mammography screening examination conducted in accordance with the European guidelines [13]. Women with a breast cancer diagnosis up to 5 years before screening invitation or mammography within the past 12 months were not eligible. Breast implants or previous TOSYMA participation were trial-specific exclusion criteria. Written informed consent was obtained from all study participants. Details of the study protocol and first results have been reported before [10–12, 14]. In brief, study recruitment took place between July 2018 to December 2020 in 17 certified screening units respectively study centers. A total of 99,689 women were randomized in a 1:1 ratio to the intervention arm, receiving digital breast tomosynthesis plus synthesized mammography (DBT + SM), or to the control arm, receiving the current screening standard, full-field DM. All data were recorded in the certified MSP documentation software MaSc (versions 5.0 and 6.0). The study was approved by the German Federal Office for Radiation Protection, and the study protocol was approved by the local ethics committee of each participating center. An independent Data Monitoring Committee reviewed the data for study participation safety.

### Imaging protocol and technical quality assurance

Twenty-seven mammography devices from five different manufacturers (FUJIFILM, GE HealthCare, Hologic Inc., IMS Giotto S.p.A, and Siemens Healthineers) were used for examinations in both trial arms. The randomization assignment determined the applied screening modus, DM or DBT + SM, and in each study center, the same mammography device was used for the examinations in both, the DM and the DBT arm.

Breasts were examined in two views (cranio-caudal (CC) and medio-lateral-oblique (MLO)). All images in both study arms were acquired using the automatic exposure control (AEC) of the respective mammography device. The AEC considers compressed breast thickness and breast density for determining the optimal radiation exposure parameters (kV, filtration, mA) as both are closely related to the required radiation exposure, i.e., the AGD.

In the DM arm, the national diagnostic reference value for the median AGD was 2.0 mGy, while a study reference value of 2.4 mGy, related to 53 mm breast thickness, had been specifically predefined in the DBT + SM arm by the Federal Office for Radiation Protection. All mammography devices used in the study centers were subjected to the national quality standards as well as to the European guidelines for breast cancer screening [13] and met all legal and technical requirements. The systems were also subjected to an extended quality assurance of the image quality and an additional stability test of the AEC. The extended quality assurance was developed specifically for this study. System software upgrades, potentially influencing the AEC and post-processing, were not allowed during the recruitment phase of TOSYMA.

### Technical data

The present analysis of radiation exposure used the AGD values as calculated by each mammography device. In general, the AGD is calculated by manufacturer-specific algorithms from the incident air kerma with correction factors for the X-ray spectrum and the absorption properties of the breast [15, 16]. The corresponding parameters including compression force and resulting breast thickness were automatically stored in the DICOM header by the device systems and recorded in the MaSc software. In case of problems with the automated documentation, a manual entry in MaSc was possible. Only CC and MLO projections were included in the analyses, special projections (e.g., partial exposures, medio lateral views) were not considered. All data were subjected to a plausibility check and AGD values below 0.05 mGy or above 15 mGy as well as compressed breast thicknesses below 2 mm and above 160 mm were excluded from the analyses.

### Medical data

The breast densities of each participant were visually categorized based on independent assignments carried out by each reader and were recorded during the independent double-reading process of each examination [13]. In total, the independent double reading was performed in both study arms by 83 qualified mammography-screening readers (12 readers with 2–4 years of experience, 22 readers with 5–9 years of experience, and 49 readers with at least 10 years) [11]. The classification criteria of the 5th edition of the Breast Imaging and Reporting Data System (BI-RADS) (A = almost fatty; B = scattered areas of fibroglandular density; C = heterogeneously dense; D = extremely dense) were applied. For study purposes, in cases of density discrepancies between two readers, the higher density category was assigned [17]. “Non-dense” breasts comprised women from categories A and B, and “dense breasts” from categories C and D.

### Statistical methods

Age of the study participant (years), compression force (N), compressed breast thickness (mm), and AGD (mGy), were analyzed across the four breast density categories in each trial arm. Data are presented as crude mean values and their standard deviations (SD) and compared by breast density and study arm. The AGD differences between study arms, derived from adjusted mean difference values with 95% confidence intervals, were calculated from a linear mixed regression model with the study arm as an independent variable, separated for different subgroups of breast density (dense and non-dense) and for two groups (below or equal and above the median) of breast thickness. To account for clustered data (correlation within centers) we added a random effect with a working correlation matrix with a compound symmetry structure.

This paper contains results of an unplanned, exploratory subanalysis after the evaluation of the first primary study hypothesis of the randomized TOSYMA trial, comparing the detection rate of invasive breast cancers in women receiving DBT + SM with women getting the DM screening, which has been completed [10]. Therefore, in this subanalysis, we follow widely accepted statistical recommendations [18] and refrain from significance testing using a predefined threshold for *p*-values (such as < 0.05). We rather present ranges of values in the form of 95% confidence intervals, which contain parameter estimates that are highly compatible with the study data and the assumptions underlying the statistical models [19]. Consequently, corrections for multiple tests were not applied.

Results are based on analyses following the as-treated principle, i.e., women were evaluated based on the screening method they actually received.

### Results

**Fig. 1:**
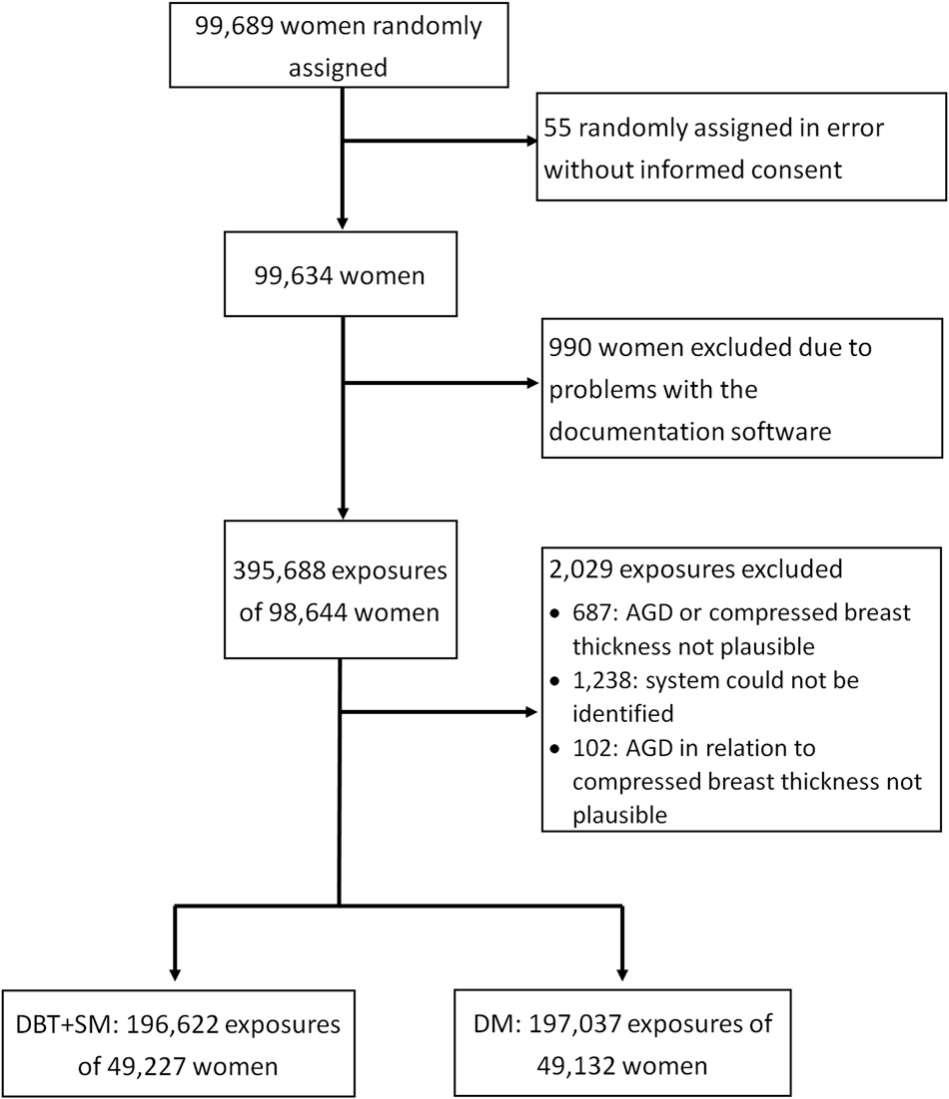
Flowchart of radiation exposures and numbers of women in this TOSYMA subanalysis. AGD, average glandular dose; DBT + SM, digital tomosynthesis plus synthesized mammography; DM, digital mammography

From a total of 99,689 trial participants, the exposure data of 990 participants were not properly recorded in the screening software and therefore excluded; a further 2029 exposures were excluded after plausibility checks. A total of 196,622 exposures from 49,227 women in the intervention arm (DBT + SM) and 197,037 exposures from 49,132 women in the control arm (DM) were available for this subanalysis (Fig. 1).

An overview of the baseline characteristics of the study population shows that the numbers of exposures per age group were similar in both study arms, resulting in an almost equal age distribution with a median age of 57 years. We noted, however, modest differences in the frequencies of the breast density categories as women with extremely dense breast tissue (category D) were more common in the DBT + SM arm (3916/49,029 = 8.0%) than in the DM arm (2611/49,001 = 5.3%) (Table 1). Furthermore, the numbers of exposures and of exposed women, respectively, per mammography system type from different manufacturers were closely similar in the intervention and the control arm (Table 2).

**Table 1:** Numbers of radiation exposures and numbers of women in the two arms of the randomized TOSYMA trial, by age group and breast density categories

	Digital mammography (DM)	Digital breast tomosynthesis plus synthesized mammography (DBT + SM)
	No. of exposures (no. of women)	No. of exposures (no. of women)
Total	197,037 (49,132)	196,622 (49,227)
Age group (years)		
50–54	72,814 (18,154)	72,283 (18,100)
55–59	49,753 (12,403)	49,949 (12,510)
60–64	43,135 (10,758)	43,717 (10,935)
65–70	31,335 (7817)	30,673 (7682)
Breast density^a^ (BI-RADS 5th edition)		
A	17,775 (4402)	17,382 (4343)
B	93,075 (23,193)	88,286 (22,096)
C	75,609 (18,891)	74,554 (18,674)
D	10,447 (2611)	15,617 (3916)
Missing^b^	131 (35)	783 (198)

*BI-RADS* Breast Imaging Reporting and Data System, *ACR* American College of Radiology

BI-RADS 5th edition [17]: A: almost entirely fatty, B: scattered areas of fibroglandular density, C: heterogeneously dense, which may obscure small masses, and D: extremely dense, which lowers the sensitivity of mammography

^a^ The highest breast density category of the independent double reading was used per examination

^b^ Missing evaluation AGD documentation or missing breast density documentation

**Table 2:** Numbers of radiation exposures and numbers of women in the two arms of the randomized TOSYMA trial, by vendor and system type

Manufacture	Mammography system type	Digital mammography (DM)	Digital breast tomosynthesis plus synthesized mammography (DBT + SM)
		No. of exposures (no. of women)	No. of exposures (no. of women)
Fujifilm	Amulet Innovality	19,968 (4942)	19,902 (4951)
IMS Giotto	Class Tomo	15,706 (3940)	15,741 (3955)
Hologic	Lorad Selenia 3Dimensions	21,641 (5403)	21,492 (5384)
Hologic	Lorad Selenia Dimensions	80,174 (19,989)	80,192 (20,050)
Siemens	MAMMOMAT Inspiration	13,539 (3374)	13,451 (3384)
Siemens	MAMMOMAT Revelation	25,450 (6353)	25,400 (6407)
GE Medical Systems	Senographe Essential	20,559 (5131)	20,444 (5097)

**Fig. 2:**
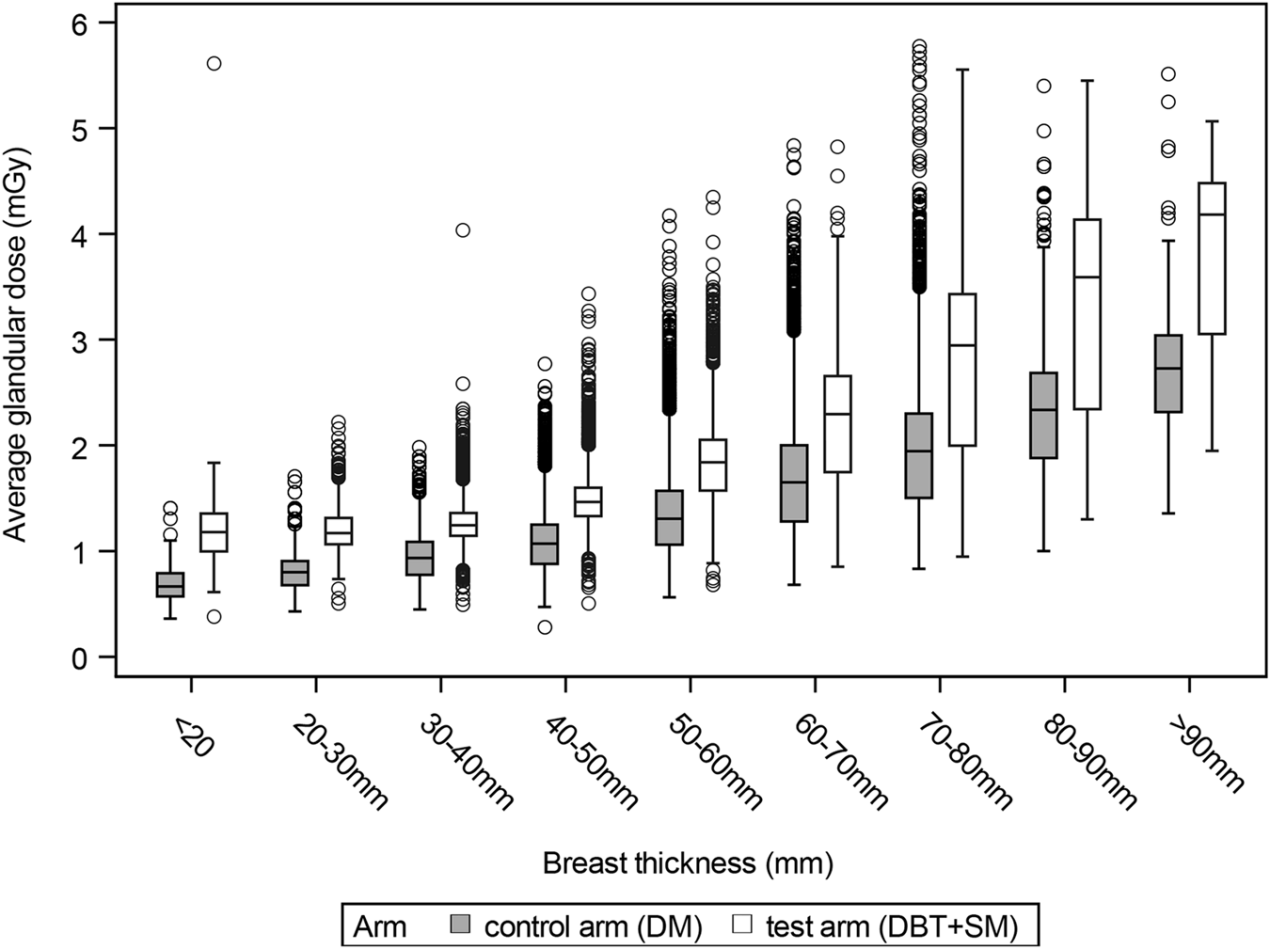
Association of average glandular dose (AGD) with breast thickness, by study arm, among participants of the TOSYMA subanalysis

The mean breast thickness was inversely related to breast density in both study arms—as were the women’s mean age and the breast compression force (Table 3). Likewise, the AGD was strongly and positively related to the mean breast thickness in both arms (Fig. 2). Interestingly, however, the AGD varied differently with breast density in the study arms (Table 4): In the DM arm, the mean AGD was almost constant at a level of 1.46 to 1.51 mGy across the density categories A to D. This contrasted with the DBT + SM arm where the mean AGD decreased from 2.41 mGy (category A), over 2.11 mGy (B) and 1.93 mGy (C) to 1.89 mGy in category D. Comparing the applied radiation doses between the study arms, the mean AGD in the DBT + SM was higher than in the DM arm by 0.94 mGy in category A, by 0.65 mGy in category B, by 0.42 mGy in C and by 0.39 mGy in category D (Table 4). Thus, in relative terms, the AGD with DBT + SM was higher than with DM by 64.4% in women from category A, by 44.5% in category B, by 27.8% in category C, and by 26.0% in category D. The mean AGD in women with non-dense breasts (i.e., category A plus B) was 0.70 mGy higher in the DBT + SM group (2.16 mGy) than in the DM group (1.46 mGy), a relative elevation of 47.9%, while in women with dense breasts (category C plus D) the mean AGD was 0.41 mGy higher with DBT + SM (1.92 mGy) than in the DM group (1.51 mGy), a relative elevation of 27.2% (Table 4). The stratification of the two groups non-dense (A + B) and dense breasts (C + D) into two breast thickness categories of non-thick breasts (≤ median 59 mm) and thick breasts (> median 59 mm), showed only slight differences of the mean AGD depending on breast density assessment within the same breast thickness stratification in the DBT + SM arm (breast density A + B non-thick 1.57 mGy, C + D non-thick 1.61 mGy, A + B thick 2.51 mGy and C + D thick 2.50 mGy). In the DM arm, the mean AGD varied comparable on a lower AGD level, e.g., the mean AGD of women with non-dense breasts and a compression thickness > 59 mm was higher than that of women with dense breasts and a compression thickness ≤ 59 mm (Table 5).

**Table 3:** Mean values (and standard deviation, SD) of breast thickness, age, and compression force, by breast density categories in the two arms of the randomized TOSYMA trial

Breast density^a^ (BI-RADS 5th edition)^b^	Mean (SD)		
	Age (years)	Compression force (*N*)	Breast thickness (mm)
Digital mammography (DM)			
A (fatty)	58.8 (5.60)	113.8 (30.5)	67.0 (10.78)
B (scattered)	58.2 (5.62)	108.7 (30.2)	61.0 (12.15)
C (heterogeneously dense)	57.1 (5.56)	102.6 (27.0)	54.1 (13.09)
D (extremely dense)	55.9 (5.37)	98.2 (24.1)	45.6 (13.85)
Total	57.7 (5.63)	106.3 (29.0)	58.0 (13.49)
Digital breast tomosynthesis plus synthesized mammography (DBT + SM)			
A (fatty)	58.8 (5.64)	113.2 (28.6)	67.6 (11.20)
B (scattered)	58.3 (5.60)	108.5 (26.4)	61.4 (12.16)
C (heterogeneously dense)	57.2 (5.53)	102.5 (25.3)	54.8 (12.99)
D (extremely dense)	55.8 (5.33)	98.1 (24.3)	47.4 (13.81)
Total	57.7 (5.61)	105.8 (26.4)	58.3 (13.54)

^a^ The highest breast density category of the independent double reading was used per examination

^b^ *BI-RADS* Breast Imaging Reporting and Data System

**Table 4:** Crude mean values of the average glandular dose (AGD) with (standard deviation, SD) and adjusted differences of AGD means (with 95% confidence intervals), by breast density categories in the two arms of the randomized TOSYMA trial

	Mean (SD) AGD (mGy)		Mean (SD) AGD (mGy)	AGD difference^e^ (mGy)	95% CI of the AGD difference
**Breast density**^**a**^ **BI-RADS**^**b**^ **(5th edition)**		**DM**^**c**^	**DBT** + **SM**^**d**^	**(DBT** **−** **DM)**	
A (fatty)		1.46 (0.53)	2.41 (0.93)	0.94	0.92; 0.99
B (scattered)		1.46 (0.55)	2.11 (0.77)	0.65	0.64; 0.67
C (heterogeneously dense)		1.51 (0.64)	1.93 (0.65)	0.42	0.40; 0.43
D (extremely dense)		1.50 (0.70)	1.89 (0.63)	0.39	0.36; 0.42
A + B (non-dense)		1.46 (0.55)	2.16 (0.81)	0.70	0.69; 0.71
C + D (dense)		1.51 (0.64)	1.92 (0.65)	0.41	0.40; 0.42
Total		1.48 (0.59)	2.05 (0.75)	0.57	0.56; 0.58

^a^ The highest breast density category of the independent double reading was used per examination

^b^ *BI-RADS* Breast Imaging Reporting and Data System

^c^ *DM* digital mammography

^d^ *DBT* *+* *SM* digital breast tomosynthesis plus synthesized mammography

^e^ Adjusted differences, and their 95% CIs, were calculated from a linear mixed model

**Table 5:** Crude mean values of the average glandular dose (AGD) with (standard deviation, SD) stratified by breast density and breast thickness in the two arms of the randomized TOSYMA trial

	Mean (SD) AGD (mGy)	Mean (SD) AGD (mGy)
**Breast density**^**a**^ **BI-RADS**^**b**^ **(5th edition)**	**DM**^**c**^	**DBT** + **SM**^**d**^
A + B (non-thick^e^)	1.08 (0.30)	1.57 (0.36)
A + B (thick^f^)	1.70 (0.53)	2.51 (0.80)
C + D (non-thick^e^)	1.24 (0.41)	1.61 (0.37)
C + D (thick^f^)	2.04 (0.68)	2.50 (0.66)

^a^ The highest breast density category of the independent double reading was used per examination

^b^ *BI-RADS* Breast Imaging Reporting and Data System

^c^ *DM* digital mammography

^d^ *DBT* *+* *SM* digital breast tomosynthesis plus synthesized mammography

^e^ Non-thick defines a breast thickness lower or equal 59.0 mm (59.0 mm was the median breast thickness)

^f^ Thick refers to a breast-thickness greater than 59.0 mm

**Fig. 3:**
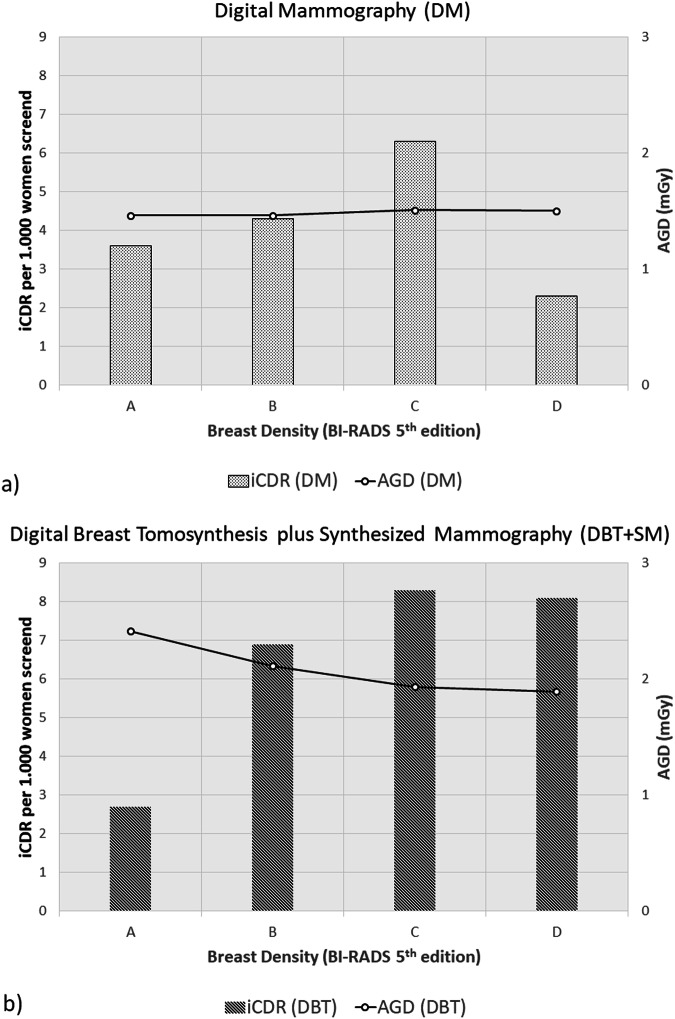
Invasive breast cancer detection rates (iCDR) and average glandular dose (AGD) in the four breast density categories: digital mammography (**a**) and digital tomosynthesis plus synthesized mammography (**b**)

Figure 3 reiterates the previously published [11] invasive cancer detection rates (iCDR) in women screened with DM (3.6 per 1000 (category A), 4.3 per 1000 (B), 6.1 per 1000 (C), and 2.3 per 1000 (D)) and the iCDR in the DBT + SM arm (2.7 per 1000 (A), 6.9 per 1000 (B), 8.3 per 1000 (C) and 8.1 per 1000 (D)). The figure reveals that, while the iCDR was raised with DBT + SM screening specifically in women with extremely dense breasts (category D), this is achieved with a radiation dose that was less elevated than in any of the three other density categories. In women with dense breasts (category C plus D) the iCDR was 5.6 for the DM group and 8.3 in the DBT + SM arm. For women with non-dense breasts (category A plus B) the iCDR was 4.2 (DM) compared to 6.2 (DBT + SM).

## Discussion

The present subanalysis of the TOSYMA trial related mammographic radiation exposure to iCDR in a screening setting that compares DBT + SM with DM. It confirmed that the radiation exposure with DBT + SM screening generally operates on a higher mean AGD level than DM. However, the relative elevation of the AGD was appreciably lower in women with dense breasts (+ 27.2%) than in those with non-dense breasts (+ 47.9%). Despite the lower radiation exposure differences, the iCDR was higher with DBT + SM than DM in women with dense breasts but not in those with non-dense breasts (Fig. 3).

The inverse association between breast density and breast thickness as observed in this TOSYMA subanalysis has been seen in other studies before. Moshina et al [20] found a negative correlation between compression breast thickness and compression force with percentage breast density assessed by automated software. This association had already been reported in phantom studies before [21, 22] and was confirmed by smaller clinical studies [23] as well as studies on larger screening populations [24, 25].

In fact, technical studies of the factors affecting radiation exposure in DBT and DM screening [25, 26] revealed that the DBT mode is more sensitive to breast thickness than the DM mode and that the AEC in the DBT mode is primarily adjusting the dose in relation to the breast thickness. The stronger dependence of DBT on breast thickness results mainly from the different X-ray spectra (kV and filters) [27]. Osteras et al reported a dose increase depending on breast thickness for DBT from 1.28 mGy (20–29 mm breast thickness) to 3.71 mGy (80–89 mm breast thickness). This corresponds to an increase of about 190% and is consistent with the increase observed in the TOSYMA study (1.19 mGy to 3.59 mGy; 200% increase, Fig. 2). It should be noted that TOSYMA is a multi-vendor study, which may explain slightly different absolute value ranges of the AGD between the studies. Moreover, the dose increase of performing DBT in dense breasts was appreciably lower than in fatty breasts [25]. These results are in line with the findings in this study and provide a plausible explanation for why the lower breast thickness in dense breasts is accompanied by a lower mean AGD in DBT but not in DM screening examinations.

The frequencies of breast density categories were comparable in the two TOSYMA trial arms but we noted that the frequency of visually assessed extremely dense breast tissue (category D) was somewhat higher with DBT + SM than with DM imaging (Table 1). A recent study using data from one mammography system manufacturer found that visual breast density assessment from synthesized mammograms was comparable to that of digital mammograms with an agreement of 80.3% and a kappa-value of 0.73 [28]. However, it has also been reported that texture analyses differed between DBT systems from different vendors and across breast density categories: this might have an impact on density assessment [29]. Therefore, the density assessment on a synthesized mammogram may potentially differ from that on DM images in a manufacturer-dependent manner. As the numbers of exposed women varied considerably between the mammography systems (Table 2), a potential manufacturer-dependent influence on the frequency of the density category assessment with SM cannot be completely ruled out.

In this report, mean values of the AGD are used for statistical reasons, as they allow the use of model-based adjustments for within-center correlations (clustering). As the AGD values show a skewed distribution, the mean values are mildly higher than the median AGD values of 1.86 mGy for DBT + SM and 1.36 mGy for DM reported from TOSYMA before [10]. As noted in the section on statistical methods, we refrained from presenting *p*-values for the comparisons between study arms and, in doing so, adhered to the recommendations of the American Statistical Association of 2016 which stated: “*p*-values do not measure the probability that a hypothesis is true” [18]. Factors such as study design, background evidence, and quality of the data are indispensably required to appraise the credibility and clinical relevance of study findings [19]. Therefore, we present here point estimates of the differences of the mean AGD in women from the four strata of breast density in both study arms including confidence intervals which reflect the high precision of the parameter ranges compatible with the TOSYMA data. Of note, the median AGD, overall and in each density stratum, were all below the stipulated study reference value of 2.4 mGy and the national reference value of 2.5 mGy for DBT [30]. Mean AGD values were highest and reached the national reference value in women having breast thick breasts (> 59 mm compression thickness), comparable with non-dense as well as dense breasts. With DM the AGD reached 2 mGy only in the subgroup of women with thicker and denser breasts. AGDs were higher for women with thicker, non-dense breasts than for women with dense breasts and thinner compressed breast thickness. These findings may guide explanations in individual cases of exposure levels above the reference values.

Considering the benefit-risk ratio, the benefit of a screening procedure is usually compared to the radiation-related risk, i.e., the lifetime attributable risk (LAR) which can be estimated based on the BEIR VII Committee’s risk models for radiation-associated breast cancer [31, 32]. Conventionally, the relative benefit of mammography screening is drawn from the results of randomized controlled trials which presently suggest a significant reduction of breast cancer mortality in women aged between 50 and 69 years who are screened using 2D mammography [33]. Weighing AGD values of DBT + SM required in women with dense breasts as compared to DM, and the associated LAR, against the higher iCDR [10, 11] and its potential survival benefits, it may be cautiously hypothesized that—despite higher radiation exposures—DBT + SM may show a more favorable benefit-risk ratio than DM screening in women with dense breasts.

A strength of TOSYMA is the large study population which was examined in 17 screening centers with screening devices from multiple manufacturers in an on-going population-wide screening program: in our view, this pragmatic “real-world” trial approach makes the results more impactful. The trial included all devices that at the time of the study start were able to produce synthetic mammograms without relevant soft- or hardware upgrades during the study recruitment. Furthermore, the number of missing values was very low.

A limitation of this subanalysis, reflected in our statistical approach, is that the analyses are exploratory and should be considered only hypothesis-generating. As established in clinical practice, the AGD data were generated and documented by the mammographic machines and values may vary by manufacturer. Furthermore, the long-term benefit of the increased invasive cancer detection by DBT screening on breast cancer mortality is yet unclear [34]. In particular, the extent of overdiagnosis has to be assessed. This aspect is addressed by the prospective follow-up of the TOSYMA study cohort, comparing the invasive interval cancer rates between the two study arms [13]. A further limitation might be the visual assessment of breast density. We have chosen the visual determination by independent double reading as this was the standard procedure in the German MSP. Results showed a similar distribution of the BI-RADS categories compared to international literature [17]. However, even with automatic breast density assessment, comparability may also be limited due to different providers of automatic breast density software, different software versions, and compatibility with different mammography manufacturers; therefore no international standard is defined [35].

In conclusion, in mammography screening with the used vendor mixture DBT + SM the AGD is positively associated with breast thickness but inversely related to breast density. Therefore, appreciably higher iCDR are achieved in women with dense breasts with only moderately raised radiation doses. This may suggest that DBT + SM screening has a more favorable benefit-risk ratio than DM screening in women with dense breasts.

## Abbreviations

AEC: Automatic Exposure Control
AGD: Average Glandular Dose
BI-RADS: Breast Imaging and Reporting Data System
CC: Cranio-caudal
DBT: Digital Breast Tomosynthesis
DM: Digital Mammography
iCDR: Invasive Cancer Detection Rate
LAR: Lifetime Attributable Risk
MLO: Medio-lateral-oblique
MSP: Mammography Screening Program
SM: Synthesized Mammography
TOSYMA: TOmosynthesis plus SYnthesized MAmmography
